# Translation and cross-cultural adaptation to Brazilian Portuguese of two brief screening tools for youth at risk of psychosis: the Prodromal Questionnaire (PQ-16) and the PRIME-Screen

**DOI:** 10.47626/2237-6089-2021-0276

**Published:** 2023-03-07

**Authors:** Ana Paula Aguiar, Angel Olider Rojas Vistorte, Henrique Teruo Akiba, Paula Oliveira, Deise Palermo Puertas Ruiz, Ary Gadelha, Rodrigo Affonseca Bressan, Pedro Mario Pan

**Affiliations:** 1 Programa de Reconhecimento e Intervenção em Estados Mentais de Risco Departamento de Psiquiatria Universidade Federal de São Paulo São Paulo SP Brazil Programa de Reconhecimento e Intervenção em Estados Mentais de Risco (PRISMA), Departamento de Psiquiatria, Universidade Federal de São Paulo (UNIFESP), São Paulo, SP, Brazil.; 2 Instituto Ame Sua Mente São Paulo SP Brazil Instituto Ame Sua Mente, São Paulo, SP, Brazil.; 3 Programa de Esquizofrenia UNIFESP São Paulo SP Brazil Programa de Esquizofrenia (PROESQ), UNIFESP, São Paulo, SP, Brazil.

**Keywords:** Psychosis, screening tools, at-risk mental states, ultra-high risk

## Abstract

**Introduction:**

Prodromal characteristics of psychosis have been described for more than a century. Over the last three decades, a variety of studies have proposed methods to prospectively identify individuals (and youth in particular) who are at high risk of developing a psychotic disorder. These studies have validated various screening instruments and made them available in several languages. Here, we describe the translation into Brazilian Portuguese and cross-cultural adaptation of two such screening tools – the Prodromal Questionnaire-16 (PQ-16) and the Prevention through Risk Identification, Management, and Education (PRIME)-Screen.

**Method:**

Two bilingual native speakers of Brazilian Portuguese translated the questionnaires from English. A native English speaker then performed back-translations into English. These back-translated versions were submitted to the original authors. They provided feedback and later approved the final versions.

**Results:**

After translation and cross-cultural adaptation, no items needed to be changed in the adapted PQ-16 and four items were revised in the PRIME-Screen. After the peer-review process, we included two suggestions in the PQ-16 to facilitate use of the tool in our cultural and social contexts. The PRIME-Screen did not need further changes.

**Conclusion:**

These new instruments can help screen Brazilian Portuguese-speaking patients who are at risk of psychosis in primary care.

## Introduction

Psychotic disorders are the ninth-leading cause of global health problems.^1-3^ People who suffer from these disorders often face delayed identification and treatment of their condition.^4^ In addition, patients often report subthreshold symptoms that are associated with mild to moderate functional impairment before their first episode of psychosis (FEP), which typically occur from weeks to months before the full-blown psychosis. Prospective assessment of subsyndromal symptoms enabled researchers to identify patients who were at higher risk of developing psychosis, which led to definition of the at-risk mental states (ARMS). Previous studies have developed brief questionnaires to screen for ARMS, but to date few have been available for low- and middle-income countries.

Some commonly reported subthreshold psychotic symptoms that characterize the ARMS syndrome include thought disorders, altered beliefs, and changes in perception and speech. In the early 1990s, an Australian group led by McGorry and Yung developed a semi-structured clinical interview to prospectively assess young people who presented such symptoms. They called their interview the Comprehensive Assessment of At-Risk Mental States (CAARMS).^5,6^ A few years later, an American group called Prevention through Risk Identification, Management, and Education (PRIME) developed their Structured Interview for Psychosis-Risk Syndrome (SIPS). This tool aimed to evaluate the same phenotype.^7^ These tools have been proven to reliably identify people who are in ARMS and have opened the door for a new field of study called early intervention in psychiatry.^8^ Comparative studies have found that both instruments perform similarly and both were able to identify people who are likely to transition from ARMS to full psychosis after two years.^9^

Early intervention has already become government policy in several developed countries (e.g., Australia and United Kingdom). However, implementing early intervention in clinical practice has proven to be a challenge. For instance, it is difficult to identify ARMS subjects correctly. To conduct CAARMS and SIPS interviews, clinicians require extensive training, specialized staff, and considerable time to administer the interviews. Consequently, using these interviews to identify people who are at risk of developing psychosis in daily clinical practice is unfeasible, especially in low-resource settings. Over time, these challenges led to development of self-reported screening tools such as the Prodromal Questionnaire (PQ) and the PRIME-Screen.^10^

The long version of the PQ has been proven to have adequate psychometric properties and a fair degree of reliability.^11,12^ It has also been translated into Portuguese.^13^ Unfortunately, this 92-item questionnaire is very long and therefore not appropriate as a screening tool for use in non-specialized settings. A Dutch group recently tested a condensed version of the tool – the PQ-16 – and demonstrated that it is a reliable and much more time-effective tool.^14^ Similarly, the North-American group developed the PRIME-Screen, a brief questionnaire based on the SIPS.^15^ The PRIME-Screen is more specific and sensitive than most other screening tools.^16^ Consequently, the PRIME-Screen has been widely adopted in studies of early detection of psychosis that use a two-stage screening process, comprising an initial brief screening procedure and a subsequent clinical interview.^17^

To date, there are no brief screening instruments for ARMS available in Brazilian Portuguese. Therefore, this study performs and describes the translation into Brazilian Portuguese and cross-cultural adaptation of the PQ-16 and the PRIME-Screen.

## Method

Prior to beginning the translation process, the authors of the present study obtained consent from the authors of the original screening tools to translate their work. We followed the guidelines proposed by Guillemin et al.^18^ for cross-cultural adaptation of psychometric instruments.

The PQ-16 has 16 items – nine items related to perceptual abnormalities, five items related to changes in thought content, and two items related to negative symptoms.^14,19^ Respondents mark their response to each item as true or false and also provide an answer on a Likert-type scale that ranks their level of associated distress on a scale from “0” (no distress) to “3” (severe distress).

The PRIME-Screen has 12 items. It is based on the positive symptom domain of the SIPS, which tracks presence of unusual mental activity such as ideas of grandeur and persecution, hallucinatory experiences, and lack of insight.^20^ Respondents indicate how much they agree or disagree with the propositions in each item regarding their experiences in the past year using a Likert-type scale ranging from “0” (definitely disagree) to “6” (definitely agree).

Our translation process included the following steps: 1) we obtained consent from the authors to translate their work; 2) two bilingual native speakers of Brazilian Portuguese independently translated the instruments into Brazilian Portuguese; 3) two psychiatrists with experience in ARMS clinical practice revised these translations; 4) a native English speaker performed back-translations into English; 5) these back-translated versions were submitted to the authors of the original scales for feedback; and 6) feedback was incorporated and a final Brazilian Portuguese version was completed.

**Figure 1:**
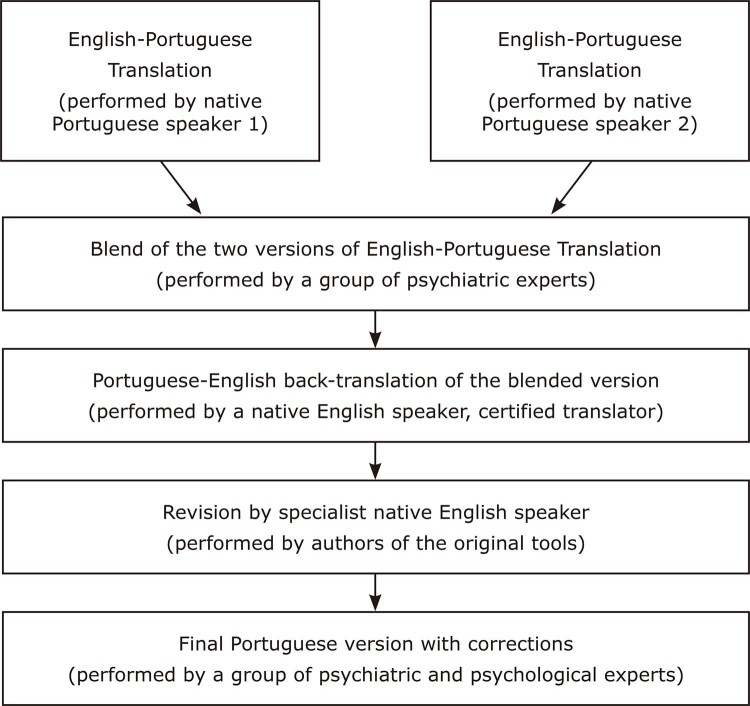
This study’s translation process.

These steps are illustrated in Figure 1.

**Figure 2:**
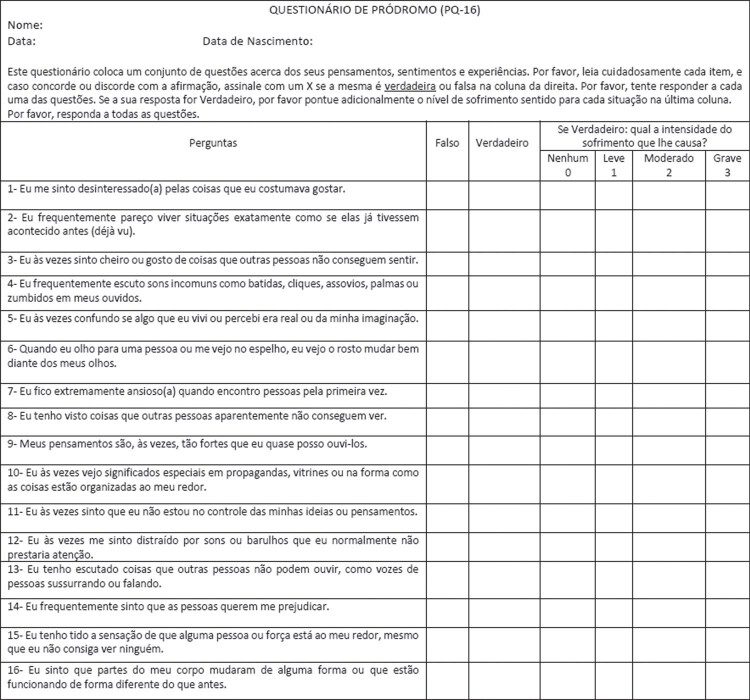
Final version of PQ-16 in Brazilian Portuguese. Permission must be requested from the original authors before using the tool.

**Figure 3:**
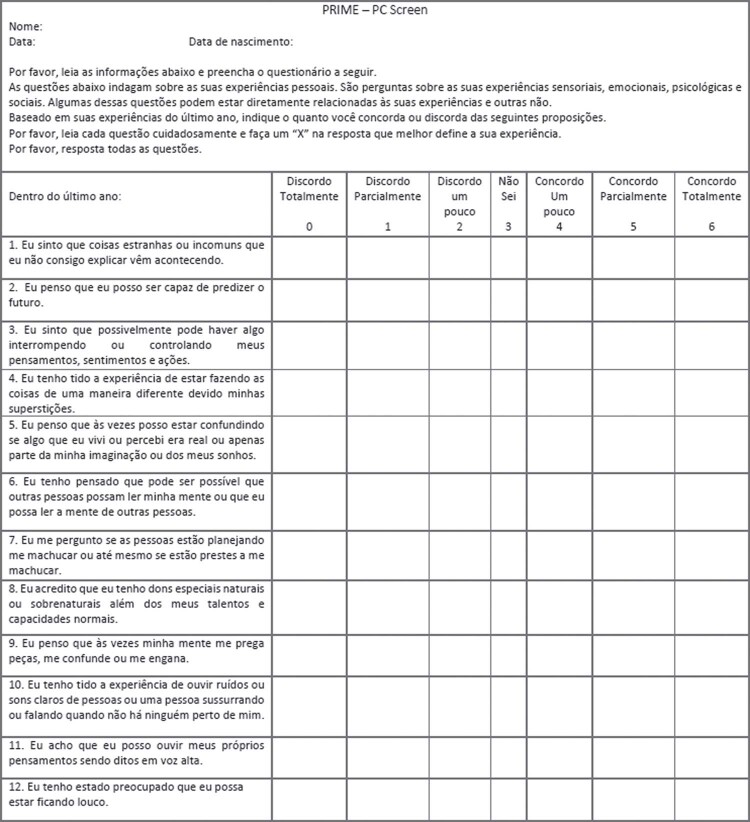
Final version of PRIME-Screen in Brazilian Portuguese. Permission must be requested from the original authors before using the tool.

As mentioned above, we sent the screening tools to the original authors after back-translating them into English. The original authors did not suggest any changes to the PQ-16 (Table 1). The original authors did point out some inconsistencies in the PRIME-Screen (Table 2); therefore, we made corrections accordingly, as shown in Tables 1 and 2. Finally, as recommended during the peer-review process of the present study, we have included two suggestions to facilitate use of the instruments in our cultural and social contexts. First, we inverted item 5 to conform to Brazilian Portuguese colloquial language, making it a less complex statement that is easier for the public to understand. Figures 2 and 3 show the final versions of the instruments.

**Table 1:** Original version of the PQ-16 in English, first translation in Brazilian Portuguese, back-translation to English by a native speaker, revision by original authors, and final revised Brazilian Portuguese version

Original version	Translation to Brazilian Portuguese	Back-translation to English	Comments from original authors	Final version
True/false no/mild/moderate/severe	Verdadeiro/falso nenhum/leve/moderado/grave	True/false none/light/moderate/heavy	None	Verdadeiro/falso nenhum/leve/moderado/grave
1. I feel uninterested in the things I used to enjoy.	1. Eu me sinto desinteressado(a) pelas coisas que eu costumava gostar.	1. I feel a loss of interest in the things I used to like.	None	1. Eu me sinto desinteressado(a) pelas coisas que eu costumava gostar.
2. I often seem to live through events exactly as they happened before (déjà vu).	2. Eu frequentemente pareço viver situações exatamente como se elas já tivessem acontecido antes (déjà vu).	2. I often seem to live situations exactly as if they had happened before (déjà vu).	None	2. Eu frequentemente pareço viver situações exatamente como se elas já tivessem acontecido antes (déjà vu).
3. I sometimes smell or taste things that other people can’t smell or taste.	3. Eu às vezes sinto cheiro ou gosto de coisas que outras pessoas não conseguem sentir.	3. I sometimes smell or taste things that others cannot.	None	3. Eu às vezes sinto cheiro ou gosto de coisas que outras pessoas não conseguem sentir.
4. I often hear unusual sounds like banging, clicking, hissing, clapping, or ringing in my ears.	4. Eu frequentemente escuto sons incomuns como batidas, cliques, assovios, palmas ou zumbidos em meus ouvidos.	4. I often hear unusual sounds like beats, clicks, whistles, clapping or buzzes in my ears.	None	4. Eu frequentemente escuto sons incomuns como batidas, cliques, assovios, palmas ou zumbidos em meus ouvidos.
5. I have been confused at times whether something I experienced was real or imaginary.	5. Eu às vezes confundo se algo que experimentei foi real ou imaginário.	5. Sometimes, I confuse if something I experienced was real or imaginary.	None	5. Eu às vezes confundo se algo que eu vivi ou percebi era real ou da minha imaginação.
6. When I look at a person, or look at myself in a mirror, I have seen the face change right before my eyes.	6. Quando eu olho para uma pessoa ou me olho no espelho, eu vejo o rosto mudar bem diante dos meus olhos.	6. When I look at someone or myself in the mirror, I see the face changing right before my eyes.	None	6. Quando eu olho para uma pessoa ou me olho no espelho, eu vejo o rosto mudar bem diante dos meus olhos.
7. I get extremely anxious when meeting people for the first time.	7. Eu fico extremamente ansioso(a) quando encontro pessoas pela primeira vez.	7. I get extremely anxious when meeting people for the first time.	None	7. Eu fico extremamente ansioso(a) quando encontro pessoas pela primeira vez.
8. I have seen things that other people apparently can’t see.	8. Eu tenho visto coisas que outras pessoas aparentemente não conseguem ver.	8. I have seen things that other people apparently cannot see.	None	8. Eu tenho visto coisas que outras pessoas aparentemente não conseguem ver.
9. My thoughts are sometimes so strong that I can almost hear them.	9. Meus pensamentos são, às vezes, tão fortes que eu quase posso ouvi-los.	9. My thoughts are sometimes so strong that I can almost hear them	None	9. Meus pensamentos são, às vezes, tão fortes que eu quase posso ouvi-los.
10. I sometimes see special meanings in advertisements, shop windows, or in the way things are arranged around me.	10. Eu às vezes vejo significados especiais em propagandas, vitrines ou na forma como as coisas estão organizadas ao meu redor.	10. I sometimes see special meanings in advertisements, shop windows, or in the way things are organized around me.	None	10. Eu às vezes vejo significados especiais em propagandas, vitrines ou na forma como as coisas estão organizadas ao meu redor.
11. Sometimes I have felt that I’m not in control of my own ideas or thoughts.	11. Eu às vezes sinto que eu não estou no controle das minhas ideias ou pensamentos.	11. I sometimes feel that I am not in control of my ideas or thoughts.	None	11. Eu às vezes sinto que eu não estou no controle das minhas ideias ou pensamentos.
12. Sometimes I feel suddenly distracted by distant sounds that I am not normally aware of.	12. Eu às vezes me sinto distraído por sons que eu normalmente não prestaria atenção.	12. I sometimes feel suddenly distracted by sounds I normally would not pay attention to.	None	12. Eu às vezes me sinto distraído por sons ou barulhos que eu normalmente não prestaria atenção.
13. I have heard things other people can’t hear, like voices of people whispering or talking.	13. Eu tenho escutado coisas que outras pessoas não podem ouvir, como vozes de pessoas sussurrando ou falando.	13. I have been hearing things that other people cannot hear, such as voices of people whispering or talking.	None	13. Eu tenho escutado coisas que outras pessoas não podem ouvir, como vozes de pessoas sussurrando ou falando.
14. I often feel that others have it in for me.	14. Eu frequentemente sinto que as pessoas querem me prejudicar.	14. I often feel that people want to hurt me.	None	14. Eu frequentemente sinto que as pessoas querem me prejudicar.
15. I have had the sense that some person or force is around me, even though I could not see anyone.	15. Eu tenho tido a sensação de que alguma pessoa ou força está ao meu redor, mesmo que eu não consiga ver ninguém.	15. I have had a feeling that some person or force is around me, even though I cannot see anyone	None	15. Eu tenho tido a sensação de que alguma pessoa ou força está ao meu redor, mesmo que eu não consiga ver ninguém.
16. I feel parts of my body have changed in some way, or that parts of my body are working differently than before.	16. Eu sinto que partes do meu corpo mudaram de alguma forma ou que estão funcionando de forma diferente do que antes.	16. I feel that parts of my body have changed in some way or are working differently than before.	None	16. Eu sinto que partes do meu corpo mudaram de alguma forma ou que estão funcionando de forma diferente do que antes.

**Table 2:** - Original version of the PRIME-Screen in English, first translation in Brazilian Portuguese, back-translation to English by a native speaker, revision by original authors, and final revised Brazilian Portuguese version

Original version	Translation to Brazilian Portuguese	Back-translation	Comments from original authors	Proposed changes	Final version
Agree/disagree definitely, somewhat, slightly not sure	Concordo/discordo totalmente, mais ou menos, levemente não tenho certeza	Agree/disagree completely, somewhat, slightly I’m not sure	None	None	Concordo/discordo totalmente, parcialmente, um pouco não sei
1. I think that I have felt that there are odd or unusual things going on that I can’t explain	1. Eu sinto que coisas estranhas ou incomuns que eu não consigo explicar vêm acontecendo.	1. I feel that strange or unusual things that I cannot explain have been happening.	None	None	1. Eu sinto que coisas estranhas ou incomuns que eu não consigo explicar vêm acontecendo.
2. I think I might be able to predict the future.	2. Eu penso que posso estar dotado da capacidade de prever o futuro.	2. I think that I might be gifted with the ability to predict the future.	“Might be gifted” changes the gist of the question.	2. I think that I may be able to predict the future.	2. Eu penso que eu posso ser capaz de predizer o futuro.
3. I may have felt that there could possibly be something interrupting or controlling my thoughts, feelings, or actions.	3. Eu sinto que possivelmente pode haver algo interrompendo ou controlando meus pensamentos, sentimentos e ações.	3. I feel that there could be something interrupting or controlling my thoughts, feelings and actions.	None	None	3. Eu sinto que possivelmente pode haver algo interrompendo ou controlando meus pensamentos, sentimentos e ações.
4. I have had the experience of doing something differently because of my superstitions.	4. Eu tenho tido a experiência de estar fazendo as coisas de uma maneira diferente devido minhas superstições.	4. I have done things in a different way due to my superstitions.	None	None	4. Eu tenho tido a experiência de estar fazendo as coisas de uma maneira diferente devido minhas superstições.
5. I think that I may get confused at times whether something I experience or perceive may be real or may be just part of my imagination or dreams.	5. Eu penso que às vezes posso estar confundindo se algo que eu vivi ou percebi era real ou apenas parte da minha imaginação ou dos meus sonhos.	5. I think I can sometimes be confused if something that I lived or felt was real or just a part of my imagination or dreams.	None	None	5. Eu penso que às vezes posso estar confundindo se algo que eu vivi ou percebi era real ou apenas parte da minha imaginação ou dos meus sonhos.
6. I have thought that it might be possible that other people can read my mind, or that I can read other’s minds.	6. Eu tenho pensado que pode ser possível que outras pessoas possam ler minha mente ou que eu possa ler a mente de outras pessoas.	6. I have been thinking that it may be possible for other people to read my mind or for me to read other people’s mind.	None	None	6. Eu tenho pensado que pode ser possível que outras pessoas possam ler minha mente ou que eu possa ler a mente de outras pessoas.
7. I wonder if people may be planning to hurt me or even may be about to hurt me.	7. Eu me pergunto se as pessoas estão planejando me machucar ou até mesmo se estão prestes a me machucar.	7. I wonder if people are planning to hurt me or even if they are about to hurt me.	None	None	7. Eu me pergunto se as pessoas estão planejando me machucar ou até mesmo se estão prestes a me machucar.
8. I believe that I have special natural or supernatural gifts beyond my talents and natural strengths.	8. Eu acredito que eu tenho dons especiais ou sobrenaturais além dos meus talentos e capacidades normais.	8. I believe I have special or supernatural gifts beyond my usual skills and abilities.	Concept of “natural” is eliminated in two places in this question.	8. I believe that I have special natural or supernatural gifts beyond my normal talents or abilities.	8. Eu acredito que eu tenho dons especiais naturais ou sobrenaturais além dos meus talentos e capacidades normais.
9. I think I might feel like my mind is “playing tricks” on me.	9. Eu penso que às vezes minha mente me prega peças ou me engana.	9. I think that sometimes my mind plays tricks on me.	None	None	9. Eu penso que às vezes minha mente me prega peças, me confunde ou me engana.
10. I have had the experience of hearing faint or clear sounds of people or a person mumbling or talking when there is no one near me.	10. Eu tenho tido a experiência de ouvir ruídos ou até mesmo sons claros de pessoas como se alguém estivesse sussurrando ou falando, mesmo quando não há ninguém por perto.	10. I have had the experience of hearing noises or even clear sounds of people, as if someone was whispering or talking, even when there is no one around.	Varies a bit too much from original question.	10. I have had the experience of hearing noises or clear sounds of people or a person whispering or talking when there is no one around me.	10. Eu tenho tido a experiência de ouvir ruídos ou sons claros de pessoas ou uma pessoa sussurrando ou falando quando não há ninguém perto de mim.
11. I think that I may hear my own thoughts being said out loud.	11. Às vezes eu acho que eu posso ouvir meus próprios pensamentos sendo ditos em voz alta.	11. Sometimes, I think that I can hear my own thoughts being spoken aloud.	Please eliminate the word “sometimes”.	11. I think that I can hear my own thoughts being spoken aloud.	11. Eu acho que eu posso ouvir meus próprios pensamentos sendo ditos em voz alta.
12. I have been concerned that I might be “going crazy.”	12. Eu tenho estado preocupado que eu possa estar ficando louco.	12. I have been worried that I might be going crazy.	None	None	12. Eu tenho estado preocupado que eu possa estar ficando louco.

## Discussion

Previous studies have shown that the PQ and PRIME-Screen are among the best instruments for screening people who are at risk of developing psychosis.^16^ Early identification of ARMS facilitates early intervention and might potentially mitigate the psychosocial impact of this condition. Here, we present translations of the PQ-16 and PRIME-Screen into Brazilian Portuguese, which may constitute simple and time-effective tools to identify ARMS in low-resource settings.

Previous studies have proposed two short versions of the PQ; the PQ-B, with 21 items,^21^ and the PQ-16.^14^ Although both have been shown to be effective and reliable instruments, a recent study found that the PQ-16 outperformed the PQ-B.^22^ A cutoff of six or more symptoms in the PQ-16 exhibited a high true positive rate (87%) and high specificity (87%) when differentiating AMRS and FEP from help-seeking youth who were not at high clinical risk, according to the CAARMS.^14^ A recent review,^19^ based on four studies evaluating the diagnostic accuracy of the PQ-16, found that a total symptom score of ≥ 6 and a distress score of ≥ 9 were valid cut-offs for community-based samples. In help-seeking populations, lower scores were suggested for distress (cut-off of ≥ 8) and symptoms (cut-off of ≥ 5). For the PRIME-screen, as proposed by the original authors, any item endorsed with a score of 6 or three items endorsed with a score of 5 are considered positive for ARMS.^15^

Finally, it is important to address some limitations of the present work. First, although previous studies have validated these instruments across several languages and cultures, we suggest that the psychometric properties of these Brazilian Portuguese versions should be investigated. Second, we also suggest that the norms and cut-offs provided in previous research should be adopted with caution, since they were not investigated in Brazilian samples. Third, the original studies typically investigated samples of young people aged 17-18 years old; therefore, utilization of these instruments in other age groups, particularly younger individuals, still needs further validation. Finally, the average educational level of Brazilian youth may differ from youth in high-income countries, which may possibly impact their ability to read and understand the items and, therefore, affect the instruments’ accuracy and applicability.

## Conclusion

The Brazilian Portuguese versions of the PQ-16 and PRIME-Screen have been successfully translated and are now available for clinicians and researchers. Future studies should test their accuracy and set norms for the Brazilian population.
